# Special Considerations for Prophylaxis for and Treatment of Anthrax in Pregnant and Postpartum Women

**DOI:** 10.3201/eid2002.130611

**Published:** 2014-02

**Authors:** Dana Meaney-Delman, Marianne E. Zotti, Andreea A. Creanga, Lara K. Misegades, Etobssie Wako, Tracee A. Treadwell, Nancy E. Messonnier, Denise J. Jamieson, Workgroup on Anthrax in Pregnant and Postpartum Women

**Affiliations:** Author affiliation: Centers for Disease Control and Prevention, Atlanta, Georgia, USA

**Keywords:** Bacillus anthracis, anthrax, antibacterial agents, antimicrobial drugs, pregnancy, postpartum period, lactation, breast-feeding, bacteria, women, treatment, antibiotics, antitoxins, PEP, postexposure prophylaxis, vaccine, vaccination, *Suggested citation for this article*: Meaney-Delman D, Zotti ME, Creanga AA, Misegades LK, Wako E, Treadwell TA, et al, Workgroup on Anthrax in Pregnant and Postpartum Women. Special considerations for prophylaxis for and treatment of anthrax in pregnant and postpartum women. Emerg Infect Dis [Internet]. 2014 Feb [*date cited*]. http://dx.doi.org/10.3201/eid2002.130611

## Abstract

In August 2012, the Centers for Disease Control and Prevention, in partnership with the Association of Maternal and Child Health Programs, convened a meeting of national subject matter experts to review key clinical elements of anthrax prevention and treatment for pregnant, postpartum, and lactating (P/PP/L) women. National experts in infectious disease, obstetrics, maternal fetal medicine, neonatology, pediatrics, and pharmacy attended the meeting, as did representatives from professional organizations and national, federal, state, and local agencies. The meeting addressed general principles of prevention and treatment for P/PP/L women, vaccines, antimicrobial prophylaxis and treatment, clinical considerations and critical care issues, antitoxin, delivery concerns, infection control measures, and communication. The purpose of this meeting summary is to provide updated clinical information to health care providers and public health professionals caring for P/PP/L women in the setting of a bioterrorist event involving anthrax.

## Abstract 2

Clinical recommendations for the prevention and treatment of anthrax among pregnant women are updated.

In 2001, the United States experienced a bioterrorist attack involving *Bacillus anthracis* spore dissemination via the postal service (*1*). Anthrax continues to be a high-priority threat agent and remains a major focus of national emergency preparedness planning (*2*). As part of a comprehensive plan for anthrax readiness, it is important to plan for the needs of pregnant, postpartum, and lactating (P/PP/L) women because of their unique immunology and physiology (*3*) and the complexities of balancing maternal and fetal risks.

## Methods for Guideline Development

In August 2012, the Centers for Disease Control and Prevention (CDC), in partnership with the Association of Maternal and Child Health Programs, held a meeting titled Anthrax: Special Considerations for Pregnant and Postpartum Women to review evidence, solicit individual expert opinion, and discuss clinical recommendations for prophylaxis and treatment of anthrax specific to obstetric populations. In preparation for the meeting, 2 systematic reviews were conducted; the first summarized the worldwide experience of 20 cases of anthrax in P/PP/L women (*4*), and the second evaluated the safety and pharmacokinetics of 14 antibiotics recommended for anthrax prophylaxis and treatment (*5*). Before the meeting, the CDC convened 5 workgroups: vaccines, antibiotic prophylaxis and treatment, other treatments and clinical considerations, health care planning, and communications. Workgroup members (Technical Appendix) participated in a series of pre-meeting conference calls to determine key issues.

In August 2012, a total of 77 experts (Technical Appendix) participated in a 2.5-day meeting to discuss the proposed CDC recommendations. Following the meeting, a clinical document was created by CDC subject matter experts; review and input were provided by workgroup participants. This information updates previously published CDC guidelines for P/PP/L women (*6*,*7*) and aligns with updated recommendations for nonpregnant adults (*8*).

## General Principles of Prevention and Treatment

Although it is not clear whether pregnancy places a woman at a higher risk of acquiring any form of *B. anthracis* infection or of developing more severe disease, it is known that anthrax is associated with maternal and fetal deaths (*4*). Given the severity of anthrax, P/PP/L women should receive the same postexposure prophylaxis (PEP) and treatment regimens as nonpregnant adults (Technical Appendix Table 1), unless there are compelling reasons for these recommendations to differ. These regimens would include antimicrobial drug treatment and vaccine for P/PP/L women who have had direct exposure to anthrax spores and antimicrobial drug treatment for women with a clinical or laboratory diagnosis of anthrax.

## Vaccine

Limited data exist to guide the use of BioThrax Anthrax Vaccine Adsorbed (AVA; Emergent BioSolutions, Rockville, Maryland) in P/PP/L women. Some safety data are available through the Department of Defense (DoD), which routinely administers AVA to select US military personnel. Although pregnant military women are exempt from vaccination with AVA, inadvertent receipt of the vaccine during pregnancy has occurred (*9*). In January 2002, the Food and Drug Administration (FDA) classified AVA as Pregnancy Category D on the basis of preliminary results of an unpublished, retrospective study of infants born to women in the US military service (www.fda.gov/OHRMS/DOCKETS/98fr/05n-0040-bkg0001.pdf). The Advisory Committee on Immunization Practices subsequently reviewed all safety data available as of March 2008, including the final results of the retrospective study, and concluded that AVA is safe to administer to anthrax-exposed women during pregnancy (*7*). However, in the pre–anthrax event setting, during which risk for anthrax exposure is low, vaccination of pregnant women is not recommended. In the setting of an anthrax event that poses a high risk for exposure to aerosolized *B. anthracis* spores, pregnancy is neither a precaution nor a contraindication to vaccination (*7*). Pregnant women at risk for inhalation anthrax should receive AVA and antimicrobial drug therapy regardless of pregnancy trimester (*7*). Furthermore, there is no biologic reason to suggest that breast-feeding women or breast-fed infants have an increased risk for adverse events after vaccination with AVA. Therefore, breast-feeding is neither a precaution nor a contraindication to vaccination in the post–anthrax event setting (*7*).

The Vaccine Adverse Events Reporting System (http://vaers.hhs.gov) collects information about pregnant women who receive AVA. Among 116 reports of pregnant women inadvertently vaccinated with AVA from September 1998 through June 2012, no concerning maternal or infant safety patterns were reported. Despite the lack of data on AVA efficacy and immunogenicity in P/PP/L women, data from other inactivated vaccines administered during pregnancy have not demonstrated that being pregnant decreases vaccine effectiveness or the immune response (*10*–*12*). The extent to which maternal-fetal transfer of anthrax antibodies occurs is not known, but experience with other vaccines suggests vaccination of pregnant women may also provide some protection to newborn infants (*13*,*14*).

## Antimicrobial Drug Prophylaxis and Treatment

In addition to vaccination, antimicrobial drugs are an essential component of prophylaxis (Technical Appendix Table 1), and combination antimicrobial drug therapy is the mainstay of treatment for anthrax (Technical Appendix Tables 2, 3), with the exception of cutaneous cases without systemic involvement, which are treated with single-agent therapy (Technical Appendix Table 4). Antimicrobial drug use in pregnant women in the setting of anthrax must be viewed in the context of the high mortality risk and the benefits of treatment for the mother and fetus, as well as possible effects on the fetus resulting from the infection or from administration of antimicrobial drugs to the mother (*15*). Although safety and pharmacokinetic data for pregnant women are limited (*5*), antimicrobial drugs used for anthrax PEP and treatment for P/PP/L women are generally the same as those for nonpregnant adults (Technical Appendix Tables 1–4).

A key distinction in pregnancy is that ciprofloxacin is preferred over doxycycline for first-line anthrax PEP (Technical Appendix Table 1), despite a Pregnancy Category C rating by the FDA (*16*). Although data exist that demonstrate musculoskeletal toxicity in studies of juvenile animal (*16*), no compelling data for humans support the occurrence of sustained injury in developing bones or joints in children treated with fluoroquinolones (*17*); reviews of ciprofloxacin use during pregnancy have not demonstrated increased risks to the human fetus (*5*,*18*,*19*).

Studies of doxycycline use during pregnancy are limited; it is rated as a Pregnancy Category D drug by the FDA (*20*) on the basis of data extrapolated from the use of tetracycline in humans and animals (*21*). For tetracycline, infant dental staining, fetal growth delays, and maternal fatty liver have been demonstrated (*21*). Reviews of studies of doxycycline use among pregnant women have not demonstrated these findings (*4*,*19*,*21*). However, a small increased risk for orofacial clefts in the fetus associated with doxycycline use in early pregnancy has been reported, although the absolute risk is likely low (*5*). Given the low risk, the severity of inhalation anthrax (*1*,*22*), and the proven efficacy of doxycycline as PEP (*23*), the risk-benefit ratio would suggest that use of doxycycline may be indicated if ciprofloxacin is unavailable.

In the anthrax PEP guidance for nonpregnant adults, levofloxacin and moxifloxacin (i.e., newer fluoroquinolones) are considered alternate fluoroquinolones to use as PEP if ciprofloxacin is not available (Technical Appendix Table 1) (*8*). For pregnant women, ciprofloxacin is preferred because experience with newer fluoroquinolones is limited and retrospective data suggest that increased fetal malformation rates may occur with newer agents, such as ofloxacin (*24*). In addition, embryotoxicity has been observed in pregnant animals exposed to newer fluoroquinolones (*25*,*26*), whereas this toxicity has not been observed with ciprofloxacin (*5*,*18*). Clindamycin is also an alternative drug for PEP for adults in the event ciprofloxacin is unavailable; the criteria for its use would not differ for pregnant or postpartum women. (Technical Appendix Table 1)

If, during a bioterrorism event, the *B. anthracis* strain is determined to be penicillin-sensitive, amoxicillin may be recommended for nonpregnant adults; the recommendation would be the same for pregnant and lactating women. In previous Advisory Committee on Immunization Practices guidelines, it was recommended that P/PP/L women should be preferentially switched to amoxicillin, even when this was not recommended for the nonpregnant adult population (*7*). CDC no longer recommends preferential use of amoxicillin simply because of concerns raised by pregnancy. Thus, P/PP/L women should continue to receive ciprofloxacin unless amoxicillin is recommended as part of the nonpregnant adult PEP regimen. Although a possible association of amoxicillin exposure with an increased risk for orofacial clefts in the fetus has been suggested, the data are inconsistent, the absolute risk is low, and long-term clinical experience of amoxicillin use in pregnancy is reassuring (*5*).

The guidance for nonpregnant adults specifies that anthrax antimicrobial drug PEP should be administered for 60 days, whether recipients are unvaccinated, partially vaccinated, or fully vaccinated (Technical Appendix Table 1) (*7*,*8*). This recommendation should also apply to P/PP/L women; there is no evidence to suggest a change in the duration of antimicrobial drug prophylaxis is necessary. However, because previously published safety studies of antimicrobial drug use in pregnancy pertain to short-term use, this extended period of prophylaxis highlights the need for additional safety data among this population.

Because of physiologic changes in pregnant and postpartum women, higher antimicrobial drug doses may be required in this population (*15*). Pharmacokinetic data for anthrax PEP antimicrobials are very limited and generally inadequate for making alternate dosing recommendations during pregnancy and the postpartum period (*5*). One study reported maternal serum ciprofloxacin levels during pregnancy were lower than those for lactating women (*5*). However, increasing the dose of ciprofloxacin needs to be carefully considered, given that ciprofloxacin may accumulate in the fetal compartment, which may be a concern with the long duration needed for PEP. One limited pharmacokinetic study suggests doxycycline doses do not require adjustment during pregnancy (*5*), but additional confirmatory data are needed. Pharmacokinetic data for amoxicillin indicate that a shorter dosing interval is needed to achieve and maintain adequate serum inhibitory levels in pregnancy (*5*). However, a shorter dosing interval (e.g., every 4 hours) for an extended period (i.e., 60 days) would likely compromise PEP adherence and tolerability and argues against the use of amoxicillin as the preferred PEP drug. Antimicrobial drug prophylaxis dosing should remain the same for P/PP/L women as for nonpregnant adults until additional data become available.

Similar to prophylactic considerations, the recommendations for anthrax treatment of P/PP/L women should be the same as for nonpregnant adults (Technical Appendix Tables 2, 3). No evidence supports extending the duration of antimicrobial drug treatment for P/PP/L women. However, as for nonpregnant adults, health care providers should have a low threshold for extending the duration of antimicrobial drug treatment for severe anthrax. Pharmacokinetic data indicates that penicillin, ampicillin, or a carbapenem may require higher doses than are recommended for nonpregnant adults (*5*). Although the worldwide experience of anthrax in pregnancy is limited to 17 reported cases, cases of *B. anthracis* infection in pregnant women from the pre-antibiotic era suggest that transplacental transmission of the bacterium may occur (*4*). Therefore, >1 of the antimicrobial drugs selected for anthrax treatment should cross the placenta. Antimicrobial drugs that likely cross the placenta in adequate quantities include ciprofloxacin, levofloxacin, amoxicillin, and penicillin (*5*). Transplacental passage of clindamycin, rifampin, doxycycline, imipenem, meropenem, and chloramphenicol has also been demonstrated, but data are limited (*5*).

## Clinical Considerations

Anthrax can cause severe disease and death among P/PP/L women (*4*), but it is not known if clinical signs and symptoms differ as a consequence of the physiologic changes that accompany pregnancy. Clinical indicators of infection may be less reliable during pregnancy, as demonstrated by reported instances in which cases of anthrax in pregnant women were misdiagnosed as resulting from other causes (*4*). In addition, abnormal laboratory values that accompany the clinical indicators of anthrax, including leukocytosis, thrombocytopenia, hemolysis and coagulopathy, can mimic obstetric conditions (e.g., preeclampsia, HELLP [hemolysis, elevated liver enzymes, low platelet count], fatty liver of pregnancy, thrombotic thrombocytopenia purpura); this mimicry may present diagnostic challenges.

Suggested radiologic testing for inhalation anthrax includes chest radiography and chest computerized tomography (CT) scan (*6*,*8*,*22*). Of note, a chest radiograph may be difficult to interpret in pregnant women because of the relative cardiomegaly and elevated diaphragms seen in the later stages of pregnancy (*27*). Although a CT scan has higher fetal radiation exposure than a chest radiograph, the severity of anthrax is such that the diagnostic benefits of CT scan outweigh the risks. Ultimately, the initial radiologic evaluation of suspected anthrax in pregnant women should not differ from that of nonpregnant adults. Because of the limitations in interpreting chest radiographs for pregnant women, a thoracic ultrasound may be useful for this purpose.

Because of lower intravascular oncotic pressure, pregnant and postpartum women are at greater risk for fluid shifts than are nonpregnant adults. Blood volume and heart rate increase and systemic vascular resistance decreases during normal pregnancy (*28*), so anthrax-related volume shifts may result in more profound hypotension than would occur in nonpregnant adults. These cardiovascular changes may decrease placental blood flow and cause fetal compromise.

Obstetrical complications, such as preterm labor, fetal distress, and even fetal loss, are likely to be seen with anthrax and may serve as clinical indicators of maternal infection or worsening maternal status. Experience with other severe infectious diseases in pregnancy has illustrated that nonreassuring fetal heart rate status may be a very early indicator of cardiovascular compromise; fetal heart rate patterns may reflect inadequate uteroplacental blood flow even before overt maternal cardiovascular changes are exhibited (*28*–*30*). Preterm labor and fetal loss have been seen in cases of anthrax (*4*); therefore, obstetric monitoring is critical in the clinical management of infected pregnant patients with anthrax, as dictated by gestational age.

## Critical Care Considerations

As with any severe infection, anthrax in pregnant women should be managed in an intensive care setting, and their care should include preparation for a possible emergent delivery (*29*). Pregnant woman are at risk for intubation failure because of anatomic changes related to weight gain and increased edema that result from lower intravascular oncotic pressure (*30*). Pregnant women are also at risk for aspiration of gastrointestinal contents because of decreased gastrointestinal motility (*15*,*28*). In cases of cutaneous anthrax involving the head, neck, and upper extremities, intubation risks may be even more pronounced, and tracheostomy may be necessary (*4*,*8*).

Early and aggressive drainage of pleural effusions and ascites by chest-tube drainage, thoracentesis, and paracentesis is recommended as an adjunct to combination antimicrobial drug treatment (*6*,*8*). These procedures can be safely performed in pregnancy under ultrasound guidance. Given the recognized importance of drainage, the indications for chest-tube drainage, thoracentesis, and paracentesis should be the same for P/PP/L women as for nonpregnant adults.

## Delivery Concerns

Anthrax during pregnancy increases the likelihood of spontaneous preterm labor or medically indicated delivery (*4*). Because of this risk for preterm delivery, maternal administration of corticosteroids to improve fetal outcome should be considered in pregnant women who have *B. anthracis* infection. Dosing and gestational age for corticosteroid administration should be according to American College of Obstetrics and Gynecology guidelines (*31*).

On the basis of the risk-benefit profile for tocolytic use in pregnant women with anthrax who have or develop preterm contractions, routine tocolysis is not recommended as treatment for preterm labor. However, short-term use of tocolytics to complete a course of corticosteroids or to secure the appropriate level of neonatal care for the fetus in the event of preterm delivery may be considered and should be individualized (*31*). However, if fetal infection is suspected or confirmed, tocolysis would be contraindicated (*31*).

Limited data that predate the advent of antibiotics suggest that the fetal compartment could become infected with *B. anthracis* (*4*). When antimicrobial drug treatment is initiated early, the risk for fetal anthrax infection and/or chorioamnionitis is unknown. However, if fetal infection is suspected or diagnosed, delivery is recommended (*31*); delivery may confer a maternal therapeutic benefit as well by decreasing anthrax toxin load in the amniotic fluid and fetal compartments. Although evidence is insufficient to recommend delivery or termination of pregnancy in a stable woman being treated for anthrax solely in the hopes of improving maternal outcome, reducing the toxin burden, or preventing other complications of anthrax, delivery should be considered if a pregnant woman does not respond to antibiotic and antitoxin treatment. All products of conception should be cultured and sent for pathologic evaluation. In the event of an intrauterine or neonatal death, an autopsy should be done to determine if anthrax bacilli are present in the fetal or neonatal tissues. As a reportable disease, any case of anthrax in the United States should be reported to the CDC.

## Antitoxins

Of the 2 antitoxins available to treat disseminated *B. anthracis*, only raxibacumab is FDA approved for use (Pregnancy Category B); anthrax immune globulin (AIG) could be available under an investigational new drug protocol or emergency-use authorization (*8*). No direct evidence is available to evaluate the safety and efficacy of AIG and raxibacumab in pregnant women and their fetuses. However, a rabbit study in which supraphysiologic intravenous doses of raxibacumab were administered during gestation did not demonstrate fetal harm in these animals (*32*). Indirect evidence suggests that both AIG and raxibacumab would be expected to cross the placenta and enter breast milk (*33*,*34*). Polyclonal intravenous immunoglobulin appears to be safe when used in pregnancy for the treatment of systemic lupus erythematosis, antiphospholipid syndrome, fetal alloimmune thrombocytopenia, HIV, and congenital heart block (*34*–*39*). Experience with monoclonal and polyclonal antibodies used in the treatment of various diseases during pregnancy has been reassuring and has resulted in an FDA Pregnancy Category B or C rating for these treatments.

Pharmacokinetic data indicate that pregnancy does not have a notable effect on dosing of immunoglobulins, provided dosing is based on weight (*40*). For both monoclonal antibodies and intravenous immunoglobulins, doses are typically not adjusted for pregnancy. Given prior reassuring experience with the use of immunoglobulins in pregnancy and the potential for substantial numbers of illnesses and deaths in patients with disseminated *B. anthracis* infection, the use of AIG and raxibacumab is recommended for P/PP/L women according to the same criteria and with the same dosing schedule as those established for nonpregnant adults. Obstetric events, such as preterm labor and fetal distress, may be harbingers of clinical deterioration and may suggest earlier use of these antitoxins during pregnancy.

## Infection Control Measures

Anthrax generally does not pose a risk for person-to-person transmission (*6*,*8*). Standard infection control precautions and contact precautions for uncontained copious drainage to prevent *B. anthracis* exposure and contamination should be used (www.cdc.gov/ncidod/dhqp/pdf/isolation2007.pdf) and are no different for P/PP/L women than for the general population. Clinical management of women who deliver neonates while receiving prophylaxis or treatment for anthrax does not require mother-infant separation. Because there is no evidence for anthrax transmission through human breast milk, anthrax exposure is not considered a contraindication to initiating or continuing breast-feeding or providing expressed human milk (*7*). Women with active cutaneous anthrax lesions on the breast should avoid infant contact and not breast-feed from the affected breast until after 48 hours of appropriate antimicrobial drug therapy. *B. anthracis* has not been isolated from cutaneous lesions 48 hours after the initiation of appropriate antimicrobial drugs (*41*); therefore, breast-feeding can resume from the affected breast after these criteria are met.

## Research Gaps

Pharmacokinetic studies designed to determine dosing and to elucidate transplacental passage of antibiotics and antitoxins during pregnancy represent the highest priority research gap. Other infections, such as rickettsia, that are treated with doxycycline may provide an opportunity to gain a better understanding of safety and pharmacokinetics of this drug in pregnancy. Ex vivo modeling systems can also be used to determine appropriate dosing of antibiotics and antitoxins, and studies evaluating placental metabolism of antibiotics may be of benefit. Additional safety data on the long-term use of antimicrobial drugs would also be important to obtain. Data are needed to determine the extent to which *B. anthracis* and anthrax antibodies from active or passive immunization enter the fetal compartment. Studies of the safety and effectiveness of AVA during pregnancy and the potential barriers to vaccine use during pregnancy and the postpartum period are also needed. Given that AVA is not recommended for pregnant women in the absence of an anthrax event, these outcomes should be captured during an event. Issues related to breast-feeding, including the potential for passive immunity conferred by breast milk and the neonatal risks following exposure to cutaneous breast lesions, should also be examined. In the pre–anthrax -event setting, animal models could address many of these research gaps. During an anthrax event, a systematic approach to capturing data related to anthrax exposure and infection in P/PP/L women should be a high priority and should include reporting of obstetric and neonatal outcomes after infection and after prophylaxis with vaccine, antimicrobial drugs, and antitoxin.

## Conclusions

Obstetric health care planning for an anthrax emergency requires knowledge of the planned public health response and coordination between the medical and public health community. Plans for inpatient and outpatient care of pregnant women must be developed before an event with anthrax exposure to ensure that health systems resources can be rapidly deployed during an emergency. Health care providers, public health responders, and local, state, and national partners must work together to develop these plans, stand ready to implement them, and ensure uniformity of messages and effective communications with each other and with the general public.

Technical Appendix Treatment recommendations for anthrax and postexposure prophylaxis after exposure to *Bacillus anthracis*; members of the Workgroup on Anthrax in Pregnant and Postpartum Women.
