# Supplementary material for: Special Considerations for Prophylaxis for and Treatment of Anthrax in Pregnant and Postpartum Women
Source: Emerg Infect Dis. 2014 Feb;20(2):e130611. doi: 10.3201/eid2002.130611 (PMC3901460; doi:10.3201/eid2002.130611)
Supplement: Technical Appendix — Treatment recommendations for anthrax and postexposure prophylaxis after exposure to Bacillus anthracis; members of the Workgroup on Anthrax in Pregnant and Postpartum Women. [file 13-0611-Techapp-s1.pdf]

Article DOI: http://dx.doi.org/10.3201/eid2002.130611

Special Considerations for Prophylaxis for and Treatment of Anthrax in Pregnant and Postpartum Women

Technical Appendix

Technical Appendix Table 1. Oral Antimicrobial Post-Exposure Prophylaxis for infection with *Bacillus anthracis**

a. For all strains, regardless of penicillin susceptibility or if susceptibility is unknown	
Non-pregnant Adults:	Modifications for Pregnant Women:
**ciprofloxacin 500 mg every 12H**<br>OR<br>**doxycycline 100 mg every 12H**<br>OR<br>levofloxacin 750 mg every 24H<br>OR<br>moxifloxacin 400 mg every 24H<br>OR<br>clindamycin† 600 mg every 8H	ciprofloxacin is preferred<br>no change in dosing
OR	
b. Alternatives for penicillin-susceptible strains	
amoxicillin 1 g every 8H<br>OR<br>penicillin VK 500 mg every 6H	
Duration of Post-Exposure Prophylaxis for *Bacillus anthracis:*<br>60 d	no change in duration

***Boldface** indicates preferred agent. Alternative selections are listed in order of preference for treatment for patients who cannot take first-line treatment, or if first-line treatment is unavailable.
†Based on in vitro susceptibility data, rather than studies of clinical efficacy.

Technical Appendix Table 2. Intravenous Antimicrobial Treatment for Systemic Anthrax with Possible/Confirmed Meningitis*

Nonpregnant Adults:	Modifications for pregnant Women:
1. A Bactericidal Agent (Fluoroquinolone)	
**ciprofloxacin 400 mg every 8H**<br>OR<br>levofloxacin 750 mg every 24H<br>OR<br>moxifloxacin 400 mg every 24H	ciprofloxacin is preferred
PLUS	
2. A Bactericidal Agent (β-lactam)	
a. For all strains, regardless of penicillin susceptibility or if susceptibility is unknown	
**meropenem 2 g every 8H**<br>OR<br>imipenem† 1 g every 6H<br>OR<br>doripenem 500 mg every 8H	at least one antibiotic with transplacental passage is recommended; ciprofloxacin, levofloxacin, meropenem, ampicillin, penicillin, clindamycin, rifampin
OR	
b. Alternatives for penicillin-susceptible strains	
penicillin G 4 million units every 4H<br>OR<br>ampicillin 3 g every 6H	
PLUS	
3. A Protein Synthesis Inhibitor	
**linezolid‡ 600 mg every 12H**<br>OR<br>clindamycin 900 mg every 8H<br>OR<br>rifampin§ 600 mg every 12H<br>OR<br>chloramphenicol¶ 1 g every 6–8 H	
Duration of treatment: for ≥2–3 weeks until clinical criteria for stability are met. Patients exposed to aerosolized spores will require prophylaxis to complete an antimicrobial drug course of 60 d from onset of illness (see Technical Appendix Table 1).	No change in duration

*Systemic anthrax includes anthrax meningitis; inhalation, injection, gastrointestinal anthrax; and cutaneous anthrax with systemic involvement, extensive edema, or lesions of the head or neck. **Boldface** indicates preferred agent. Alternative selections are listed in order of preference for treatment for patients who cannot take first-line treatment, or if first-line treatment is unavailable.
† Increased risk of seizures associated with imipenem/cilastatin treatment
‡Linezolid should be used with caution in patients with thrombocytopenia, as it may exacerbate it. Linezolid use for >14 d carries additional risk for hematopoietic toxicity.
§Rifampin is not a protein synthesis inhibitor; however, it may be used in combination with other antimicrobials based on its in vitro synergy.
¶Should only be used if other options are not available, due to toxicity concerns.

Publisher: CDC; Journal: Emerging Infectious Diseases
Article Type: Online Report; Volume: 20; Issue: 2; Year: 2014; Article ID: 13-0611
DOI: 10.3201/eid2002.130611; TOC Head: Online Report

Technical Appendix Table 3. Intravenous Antimicrobial Treatment for Systemic Anthrax When Meningitis Has Been Excluded*

Non-pregnant adults	Modifications for pregnant Women:
1. A Bactericidal Antimicrobial	
a. For all strains, regardless of penicillin susceptibility or if susceptibility is unknown	
**ciprofloxacin 400 mg every 8H**<br>OR<br>levofloxacin 750 mg every 24H<br>OR<br>moxifloxacin 400 mg every 24H<br>OR<br>meropenem 2 g every 8H<br>OR<br>imipenem† 1 g every 6H<br>OR<br>doripenem 500 mg every 8H<br>OR<br>vancomycin 60 mg/kg/day IV divided every 8 h<br>(maintain serum trough concentrations of 15 – 20 µg/mL)	ciprofloxacin is preferred<br>at least one antibiotic with transplacental passage is recommended; ciprofloxacin, levofloxacin, meropenem, ampicillin, penicillin, clindamycin, rifampin
OR	
b. Alternatives for penicillin-susceptible strains	
penicillin G 4 million units every 4H<br>OR<br>ampicillin 3 g every 6H	
PLUS	
2. A Protein Synthesis Inhibitor	
**clindamycin 900 mg every 8H**<br>OR<br>**linezolid‡ 600 mg every 12H**<br>OR<br>doxycycline§ 200 mg initially,<br>then 100 mg every 12H<br>OR<br>rifampin¶ 600 mg every 12H	
Duration of treatment: for ≥2 weeks until clinical criteria for stability are met. Patients exposed to aerosolized spores will require prophylaxis to complete an antimicrobial drug course of 60 d from onset of illness (see Technical Appendix Table 1).	No change in duration

*Systemic anthrax includes anthrax meningitis; inhalation, injection, gastrointestinal anthrax; and cutaneous anthrax with systemic involvement, extensive edema, or lesions of the head or neck. **Boldface** indicates preferred agent. Alternative selections are listed in order of preference for treatment for patients who cannot take first-line treatment, or if first-line treatment is unavailable. †Increased risk of seizures associated with imipenem/cilastatin treatment
‡Linezolid should be used with caution in patients with thrombocytopenia, as it may exacerbate it. Linezolid use for >14 d carries additional risk for hematopoietic toxicity.
§A single 10–14 course of doxycycline is not routinely associated with tooth-staining.
¶Rifampin is not a protein synthesis inhibitor; however, it may be used in combination with other antimicrobials based on its in vitro synergy.

Technical Appendix Table 4. Oral Antimicrobial Treatment for Cutaneous Anthrax without Systemic Involvement*

Non-pregnant adults<br>a. For all strains, regardless of penicillin susceptibility or if susceptibility is unknown	Modifications for pregnant women
**ciprofloxacin 500 mg every 12H**<br>OR<br>**doxycycline 100 mg every 12H**<br>OR<br>**levofloxacin 750 mg every 24H**<br>OR<br>**moxifloxacin 400 mg every 24H**<br>OR<br>clindamycin† 600 mg every 8H	ciprofloxacin is preferred
OR	
b. Alternatives for penicillin-susceptible strains	
amoxicillin 1 g every 8H<br>OR<br>penicillin VK 500 mg every 6H	
Duration of Treatment:<br>60 d	No change on duration

*Recommendations are specific to cutaneous anthrax in the setting of bioterrorism. **Boldface** indicates preferred agent. Alternative selections are listed in order of preference for treatment for patients who cannot take first-line treatment, or if first-line treatment is unavailable..
†Based on in vitro susceptibility data, rather than studies of clinical efficacy.

Members of the Workgroup on Anthrax in Pregnant and Postpartum Women:

Mahmoud Ahmed University of Texas Medical Branch

Martina Louise Badell Emory University

Kristin Banks Centers for Disease Control and Prevention (CDC)

Wanda Barfield CDC

Michael Bartenfeld CDC

Rich Beigi* Magee-Women's Hospital

Tina Bhavsar* CDC

William Bower* CDC

John Bradley* American Academy of Pediatrics

Deborah Brower Health Resources and Services Administration

Renee Brown-Bryant* CDC

William Callaghan* CDC

Tegan Callahan Association of Maternal & Child Health Programs

Amanda Cohn* CDC

Ava Marie S. Conlin* Naval Health Research Center

Andreea Creanga* CDC

Deborah Dee* CDC

Gary Disbrow Biomedical Advanced Research and Development Authority

Jeffrey Ecker* Massachusetts General Hospital

Jesse Geibe CDC

Alma Gomez Association of Maternal & Child Health Programs

Susan Gorman CDC

Jacqueline Grant* State of Georgia

Violanda Grigorescu CDC

Gary Hankins* University of Texas Medical Branch

Piia Hanson Association of Maternal & Child Health Programs

Karen E, Hays* American College of Nurse-Midwives

Mary F. Hebert University of Washington

Robert Heine* Duke University

Katherine Hendricks* CDC

Jack Herrmann National Association of County and City Health Officials

Kate Howe* Association of Maternal & Child Health Programs

Mark L. Hudak* University of Florida College of Medicine -Jacksonville

Denise Jamieson* CDC

Ayten Kadanali, Umraniyae EAH, Turkey

Sedat Kadanali, Medical Park Hospital Turkey

Juliette Kendrick* CDC

Hye-Joo Kim* CDC

George Korch Office of the Assistant Secretary for Preparedness and Response

Marian McDonald CDC

Michael M. McNeil* CDC

Dana Meaney-Delman* CDC

Nancy Messonnier* CDC

Lara Misegades* CDC

Mirjana Nesin* National Institutes of Health

Stacey Parker CDC

Alison Patti, CDC

Andrew Pavia* University of Utah

Georgina Peacock* CDC

Roberta Prepas* American College of Nurse Midwives

Conrad P. Quinn* CDC

Lauren Raskin Ramos Association of Maternal & Child Health Programs

Sonja Rasmussen* CDC

Mirelys Rodriguez CDC

Paula Rosenberg CDC

Catherine Ruhl* Association of Woman's Health, Obstetric and Neonatal Nurses

Mark Sewell U.S. Army

Sean Shadomy* CDC

Jeanne S. Sheffield* University of Texas Southwestern Medical Center

Tom Shimabukuro* CDC

David Siegel National Institutes of Health

Laura Smith CDC

David Soper* Medical University of South Carolina

Helen Stallings Office of the Assistant Secretary for Preparedness and Response

Tracee Treadwell CDC

Methodius Tuuli* Washington University in St. Louis

Etobssie Wako* CDC

Lee Warner CDC

D. Heather Watts* National Institutes of Health

George Wendel Jr.* American Board of Obstetrics and Gynecology

Linda West* CDC

Amy William CDC

Melissa Willis Biomedical Advanced Research and Development Authority

Mary Wright* National Institutes of Health

Yon Yu* CDC

Laurie Zephyrin Department of Veterans Affairs

Marianne Zotti* CDC

*Designates pre-meeting work group participant.
